# Supplementary material for: WASP-mediated regulation of anti-inflammatory macrophages is IL-10 dependent and is critical for intestinal homeostasis
Source: Nat Commun. 2018 May 3;9:1779. doi: 10.1038/s41467-018-03670-6 (PMC5934380; doi:10.1038/s41467-018-03670-6)
Supplement: Supplementary file 1 — Supplementary Information [file 41467_2018_3670_MOESM1_ESM.pdf]

WASP mediated regulation of anti-inflammatory macrophages is IL-10 dependent and is critical for intestinal homeostasis

Biswas et al.

Supplemental Figure

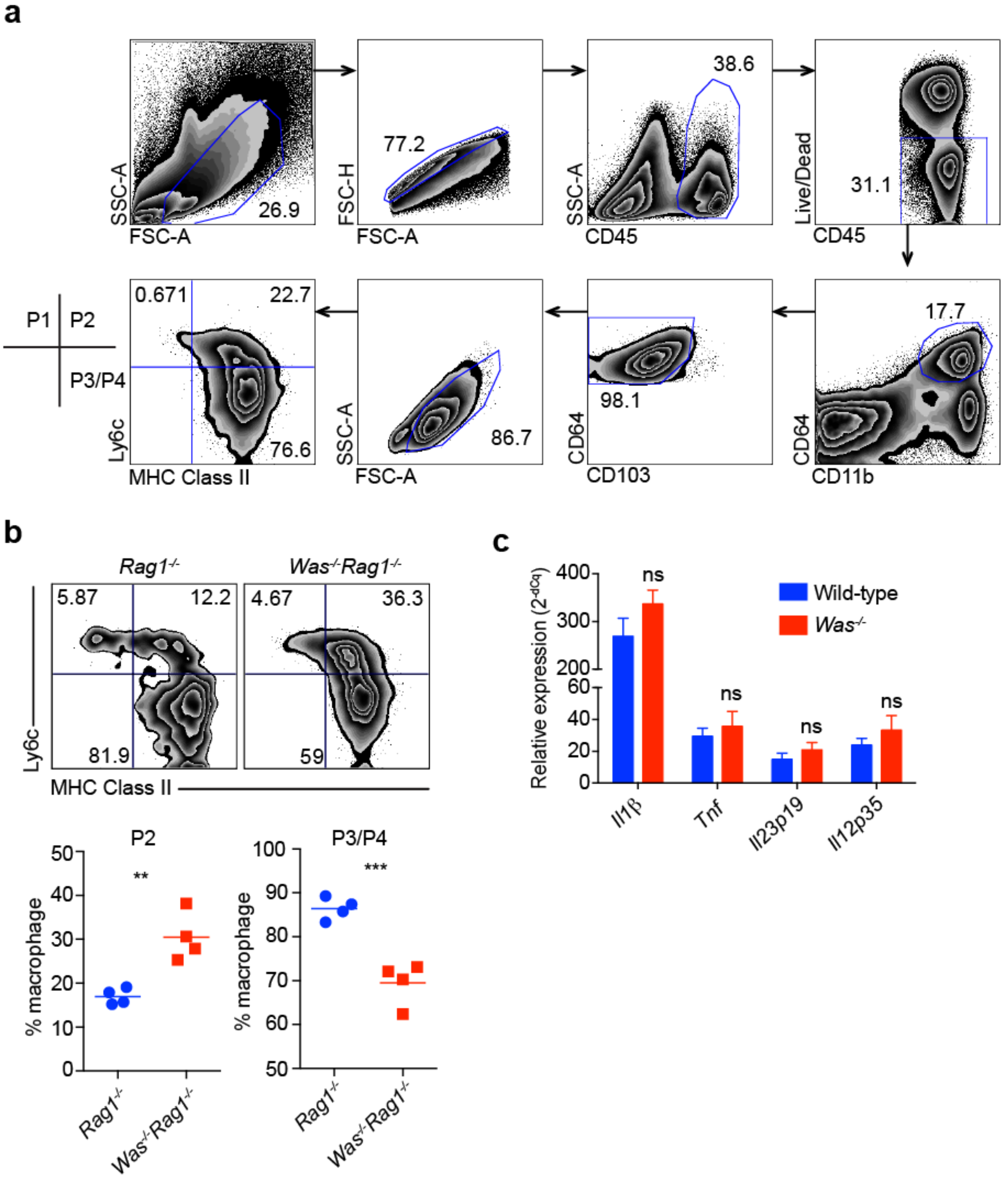

Supplementary Figure 1. Defective anti-inflammatory Mϕ in the colon of *Was*$^{-/-}$ *Rag1*$^{-/-}$mice. (a) Flow cytometry gating strategy for the analysis of lamina propria macrophages using *Was*$^{-/-}$ mice. Quadrant marking shows pro- (P2; Ly6c^{+}MHCII^{+}) and anti-inflammatory (P3+P4; Ly6c^{+}MHCII^{-}) subsets. FACS plot was representative of three independent experiments. **(b)** Flow cytometric analysis of LP macrophages in WT (wild-type) (n=4) and *Was*$^{-/-}$*Rag*$^{-/-}$ (n=4) mice at 12 weeks of age followed by quantification of pro- (P2) and anti-inflammatory (P3+P4) subsets. Macrophages were gated as live CD45^{+}CD11b^{+}CD103^{-}CD64^{+} cells. FACS plot was representative of three independent experiments. Unpaired Student's t-test was performed to determine difference between two groups. ***p* < 0.01, ****p* < 0.001. **(c)** Expression of pro-inflammatory genes in sorted P2 macrophages from WT (n=12) and *Was*$^{-/-}$ (n=12) mice. P2 cells from three mice are pooled together to increase RNA yield. Unpaired Student's t-test was performed to determine difference between two groups. *ns*: not significant.

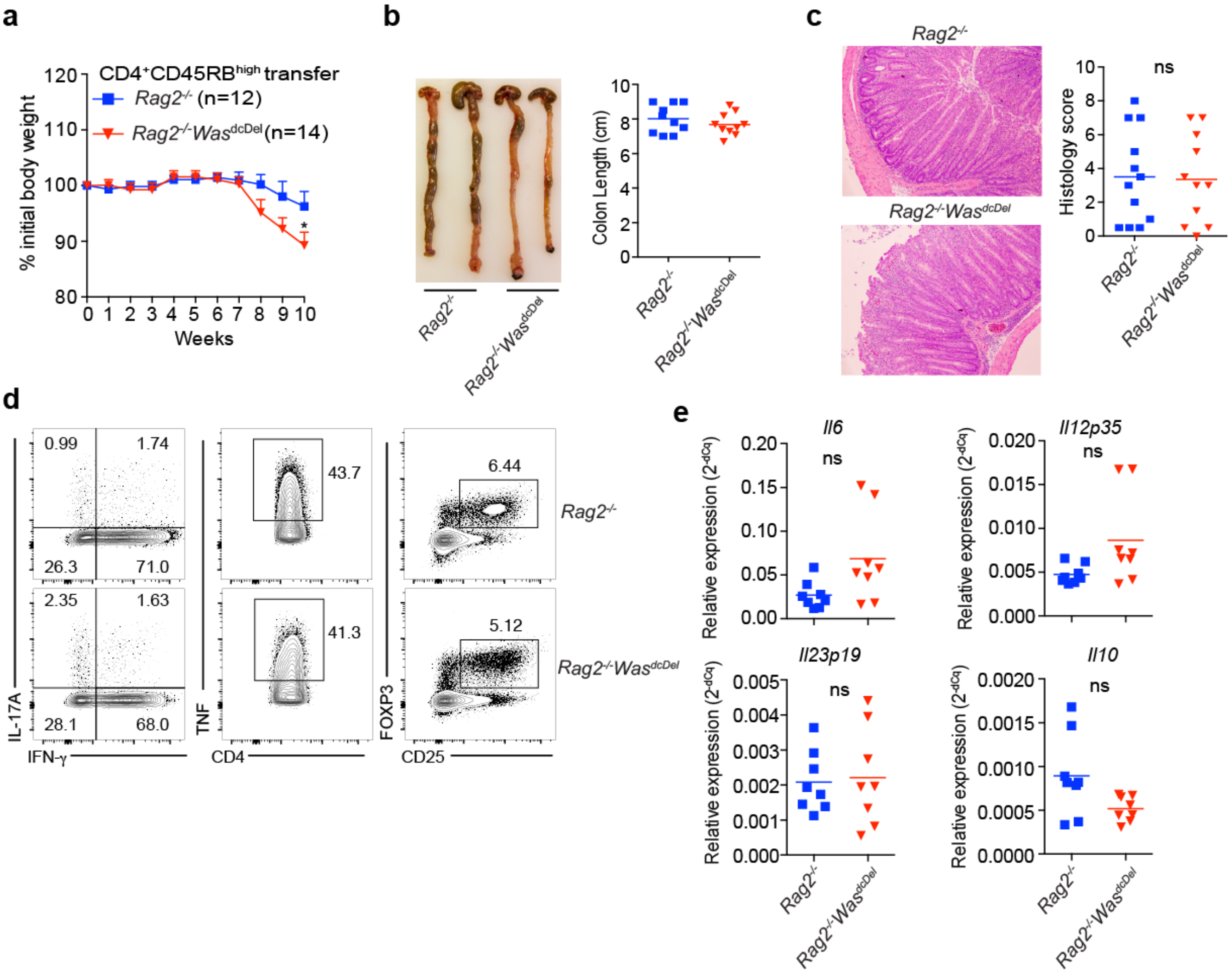

Supplementary Figure 2. DC-specific expression of WASP is not critical for the regulation of T cell transfer induced colitis. Naïve $CD4^+CD25^-CD45RB^{high}$ T cells (3-5 X 10^5) from WT (wild-type) mice were transferred intraperitoneally into *Rag2*$^{-/-}$ and *Was*^{dcDel}*Rag2*$^{-/-}$ mice to induce colitis. **(a)** % initial body weight (mean±SEM) was plotted for *Rag2*$^{-/-}$ (n=12) and *Was*^{dcDel}*Rag2*$^{-/-}$ (n=14) mice. Two-way ANOVA was used to determine statistical significance. $^*p < 0.05$. Data are cumulative of three independent experiments. **(b)** Ten weeks after transfer colon were removed and colon length was measured. *Rag2*$^{-/-}$ (n=10) and *Was*^{dcDel}*Rag2*$^{-/-}$ (n=10). Data are cumulative of three independent experiments. Unpaired Student's t-test was performed to determine difference between two groups. *ns*: not significant. **(c)** Representative photomicrographs of H&E stained colonic section and histological score of *Rag2*$^{-/-}$ (n=12) and *Was*^{dcDel}*Rag2*$^{-/-}$ (n=11) mice at 10 weeks following naïve T cell transfer. Data are cumulative of three independent experiments. Unpaired Student's t-test was performed to determine difference between two groups. *ns*: not significant. **(d)** Flow cytometry plots shows percentage of TNF^+, IFN-γ^+, IL-17A^+ and FoxP3$^+$ helper T cells in the lamina propria of *Rag2*$^{-/-}$ and *Was*^{dcDel}*Rag2*$^{-/-}$. FACS plot was representative of three independent experiments. **(e)** Cytokine expression was measured by qPCR form the colonic tissue of *Rag2*$^{-/-}$ (n=8) and *Was*^{dcDel}*Rag2*$^{-/-}$ (n=8) mice 10 weeks after T cell transfer. Data are cumulative of two independent experiments. Unpaired Student's t-test was performed to determine difference between two groups. *ns*: not significant.

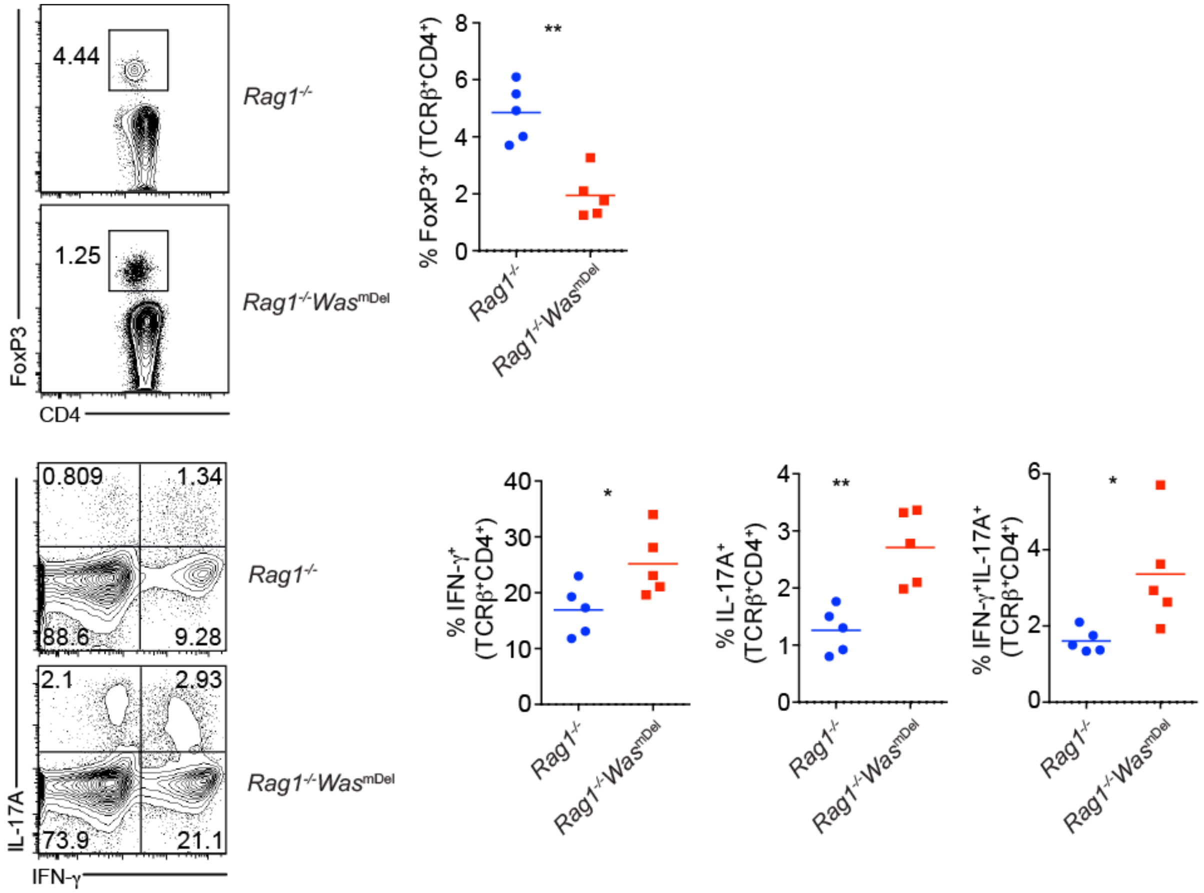

Supplementary Figure 3. Mϕ-specific expression of WASP is critical for Treg generation and regulation of Th1 and Th17 cell in MLN. Naïve $CD4^+CD25^-CD45RB^{high}$ T cells (3-5 X 10^5) from WT (wild-type) mice were transferred intraperitoneally into *Rag1*$^{-/-}$ (n= 5) and *Was*^{mDel}*Rag1*$^{-/-}$ (n=5) mice to induce colitis. Flow cytometry plots show percentage of FoxP3$^+$ (upper panel) IFN-γ$^+$ and IL-17A$^+$ (lower panel) helper T cells in the MLN of *Rag1*$^{-/-}$ and *Was*^{mDel}*Rag1*$^{-/-}$ mice at six week after T cell transfer. FACS plot was representative of two independent experiments. Unpaired Student's t-test was performed to determine difference between two groups. *$p < 0.05$, **$p < 0.01$, ***$p < 0.001$.

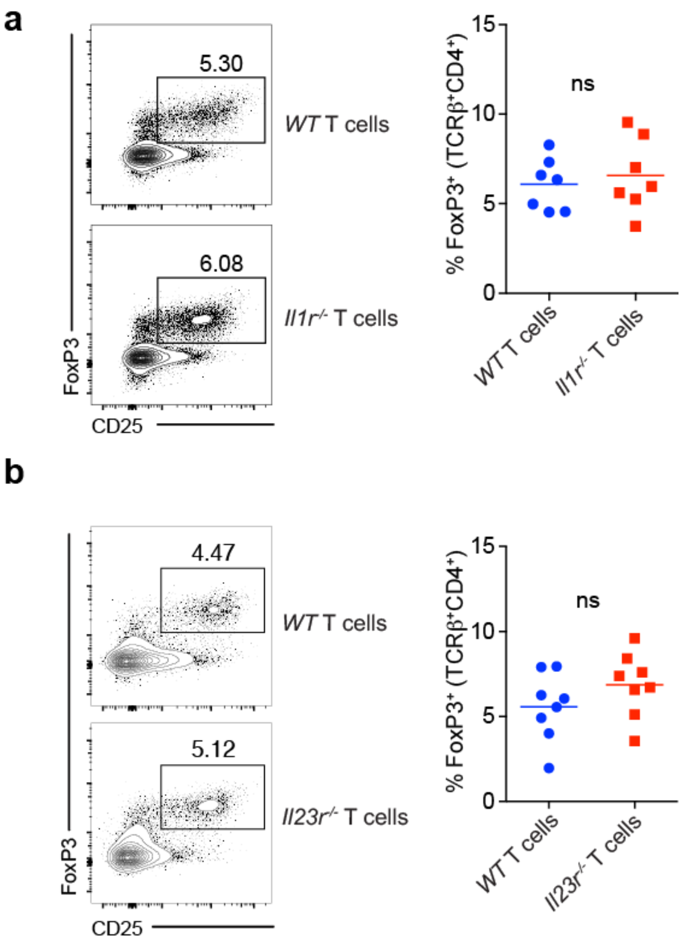

Supplementary Figure 4. Colonic Treg cell generation in *Was*^{mDel}*Rag1*$^{-/-}$ mice following *Il1r*$^{-/-}$ and *Il23r*$^{-/-}$ naïve CD4^{+} T cell transfer (a) Naïve CD4^{+}CD25^{-}CD45RB^{high} T cells (3-5 X 10^{5}) from WT (wild-type) or *Il1r*$^{-/-}$ mice were transferred intraperitoneally into *Was*^{mDel}*Rag1*$^{-/-}$ mice to induce colitis. Flow cytometry plots shows percentage of FoxP3^{+} helper T cells in the LP. Results were shown for individual mouse (WT, n=7; *Il1r*$^{-/-}$, n=7). Data are cumulative of two independent experiments. **(b)** Naïve CD4^{+}CD25^{-}CD45RB^{high} T cells (3-5 X 10^{5}) from WT or *Il23r*$^{-/-}$ mice were transferred intraperitoneally into *Was*^{mDel}*Rag1*$^{-/-}$ mice to induce colitis. Flow cytometry plots shows percentage of FoxP3^{+} helper T cells in the LP. Results were shown for individual mouse (WT, n=8; *Il1r*$^{-/-}$, n=8). Data are cumulative of two independent experiments. Unpaired Student's t-test was performed to determine difference between two groups. *ns*: not significant.

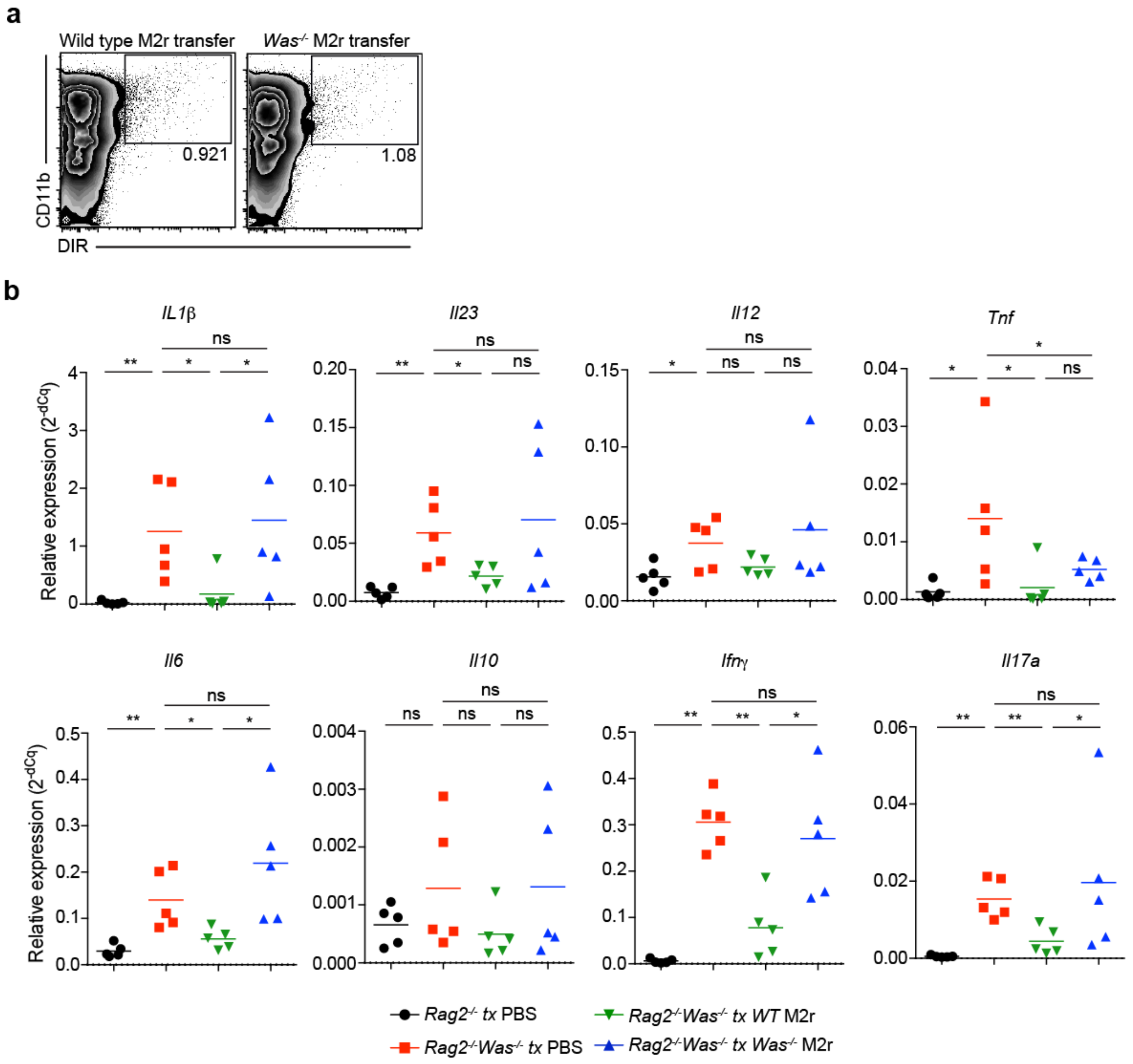

Supplementary Figure 5. Wild type M2r Mϕ restored the expression of inflammatory mediators in *Was*$^{-/-}$*Rag*$^{-/-}$ mice from T cell transfer induced colitis. (a) WT (2 X10^{6}) and *Was*$^{-/-}$ M2r (2 X10^{6}) macrophages were labelled with DIR and transferred via i.p. in *Was*$^{-/-}$*Rag1*$^{-/-}$ mice. Seven days post transfer labelled M2r macrophages were analysed in the LP. Data are representative of two independent experiments **(b)** *Was*$^{-/-}$*Rag1*$^{-/-}$ mice were transfer with PBS (n=5), WT M2r (2 X10^{6}) (n=5) or *Was*$^{-/-}$ M2r (2 X10^{6}) (n=5) macrophages one-day prior to WT CD4^{+} T cells transfer. As a control *Rag1*$^{-/-}$ (n=5) were treated with PBS and transferred with WT CD4^{+} T cells. Colonic expressions of cytokines were measured by qPCR. Results were shown for individual mouse. Data are cumulative of two independent experiments. Unpaired Student's t-test was performed to determine difference between groups. *$p < 0.05$, **$p < 0.01$, *ns*: not significant.

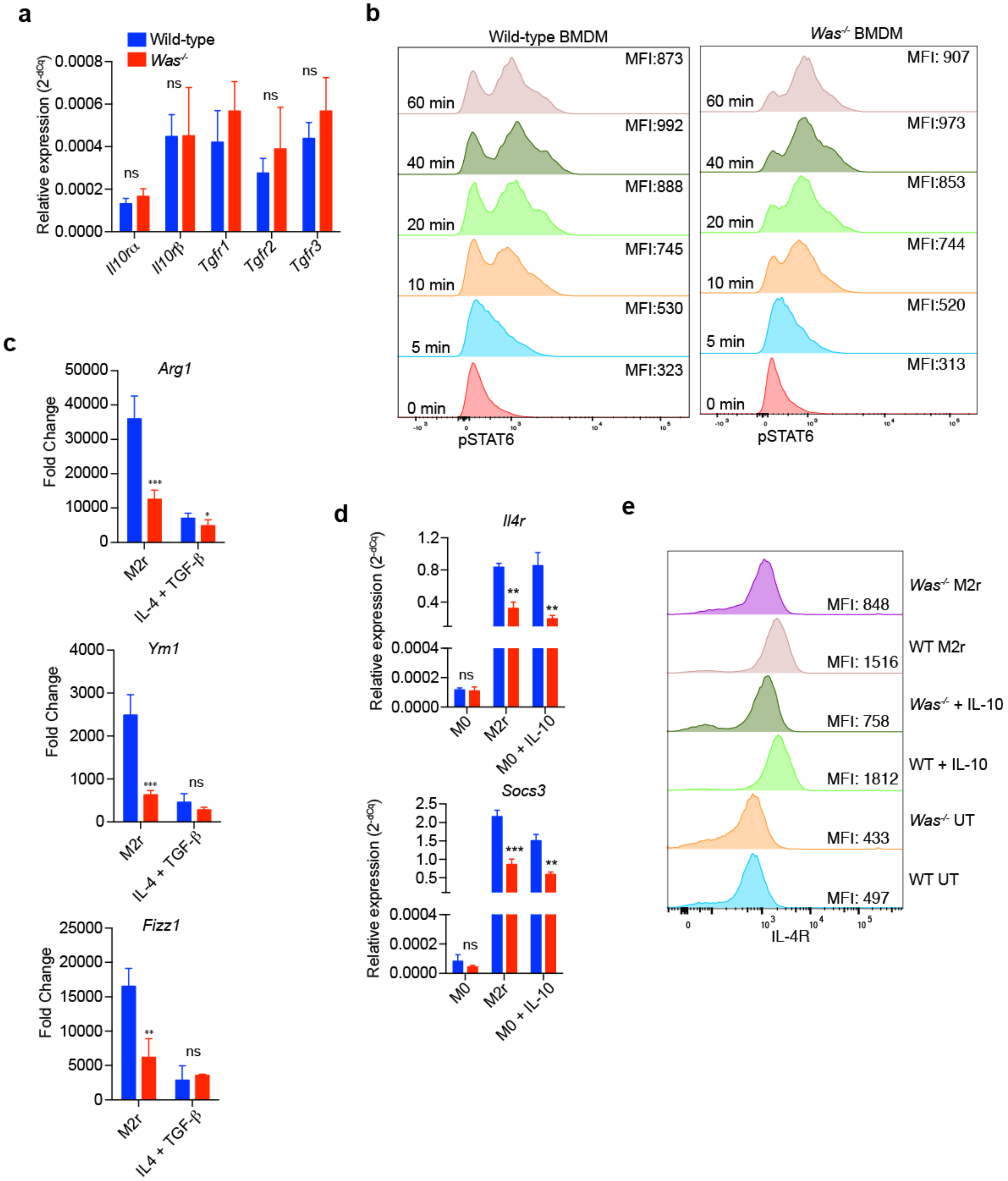

Supplementary Figure 6. IL-10 mediated regulation of anti-inflammatory macrophages is impaired in absence of WASP. (a) Expression of *Il10r* and *Tgfr* in WT (wild-type) (n=4) and *Was*$^{-/-}$ (n=4) BMDMs was analysed by qPCR at homeostasis. Data are cumulative of two independent experiments. **(b)** WT and *Was*$^{-/-}$ BMDMs were cultured in presence of IL4 (20ng/ml) for the indicated time and pSTAT6 expression was analysed by flow cytometry. Data are representative of two independent experiments. **(c)** BMDM from WT (n=4) and *Was*$^{-/-}$ (n=4) mice were cultured in presence of IL-4, IL-10 and TGF-β (M2r) or IL-4 and TGF-β, and M2 specific genes expression was analysed by qPCR. Data are cumulative of two independent experiments. BMDM from WT and *Was*$^{-/-}$ mice were left untreated (M0), cultured in presence of IL-4, IL-10 and TGF-β (M2r) or IL-10 (M0+IL-10) for 24h. (**d**) Expression of *Il4r* and *Socs3* was analysed by qPCR (WT n=4;

Was$^{-/-}$ n=4). Data are cumulative of two independent experiments. (**e**) Cell surface expression of IL-4R was examined by flow cytometry. FACS plot is representative of two independent experiments. Data shown in **a,c** and **d** are mean ± s.e.m. and P value was obtained by Student's t-test. *$p < 0.05$, **$p < 0.01$, ***$p < 0.001$, *ns*: not significant.

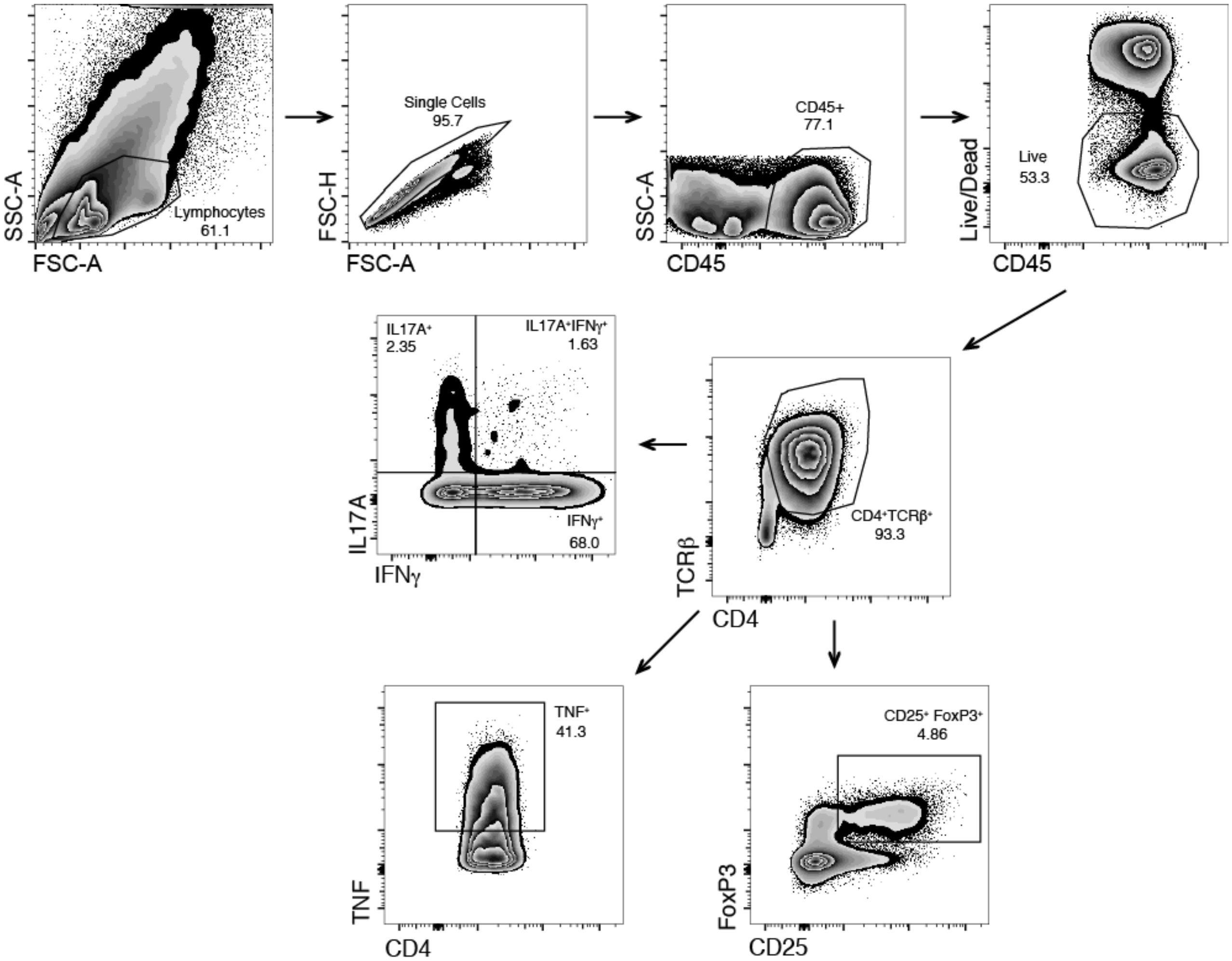

Supplementary Figure 7. Flow cytometry gating strategy for the analysis of various T cell subsets. Th1 ($CD45^{+}TCR\beta^{+}CD4^{+}INF\gamma^{+}$); Th17 ($CD45^{+}TCR\beta^{+}CD4^{+}IL17A^{+}$); Th17/Th1 ($CD45^{+}TCR\beta^{+}CD4^{+}IL17A^{+}$ $INF\gamma^{+}$); Tregs ($CD45^{+}TCR\beta^{+}CD4^{+}FoxP3^{+}CD25^{+}$). This gating strategy was used to analyse T cell subsets in Fig. 2, 3 and 5.

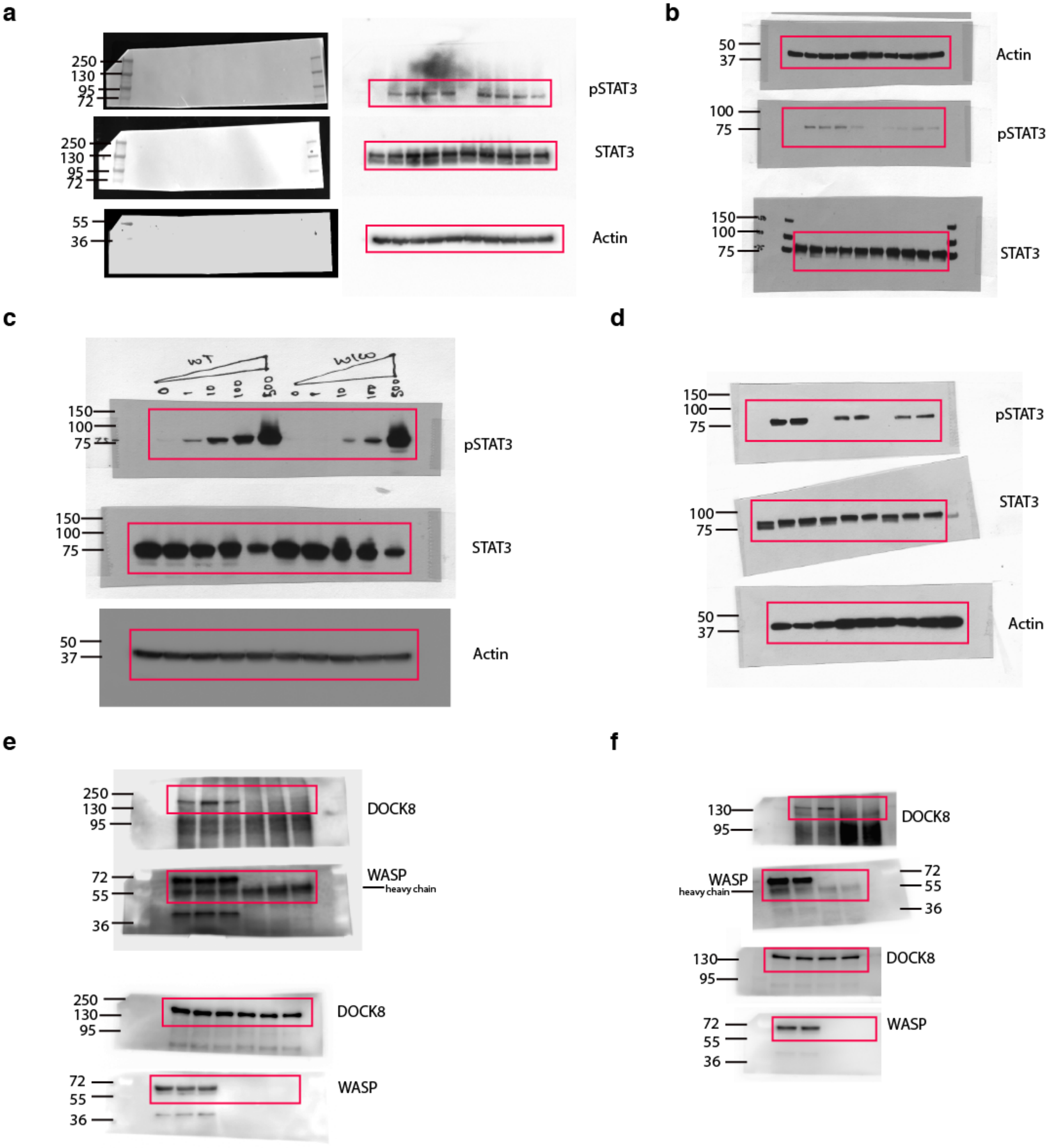

Supplementary Figure 8. Original data of immunoblots. (**a**) Immunoblot data corresponding to Fig. 6a. (**b**) Immunoblot data corresponding to Fig. 6b. (**c**) Immunoblot data corresponding to Fig. 6c. (**d**) Immunoblot data corresponding to Fig. 6d. (**e**) Immunoblot data corresponding to Fig. 6e. (**f**) Immunoblot data corresponding to Fig. 6f.

Supplementary table 1: Antibody used for flow cytometry and Western blotting

Antibody	Clone	Dilution	Source
Anti-mouse-CD45	30-F11	1:500	Biolegend
Anti-mouse-CD11b	M1/70	1:2000	Biolegend
Anti-mouse-CD11c	N418	1:200	Biolegend
Anti-mouse-CD4	RM4-5	1:400	Biolegend
Anti-mouse-CD64	X54-5/7.1	1:200	Biolegend
Anti-mouse-MHCII	M5/114.15.2	1:2000	Biolegend
Anti-mouse-Ly6c	HK1.4	1:1000	Biolegend
Anti-mouse-103	2E7	1:200	Biolegend
Anti-mouse-CD45.1	A20	1:200	Biolegend
Anti-mouse-CD45.2	104	1:500	Biolegend
Anti-mouse-CD25	PC61.5	1:200	Biolegend
Anti-mouse-TCRb	H57-597	1:400	Biolegend
Anti-mouse-FoxP3	FJK-16s	1:50	eBioscience
Anti-mouse-TNF	MP6-XT22	1:200	Biolegend
Anti-mouse-IFNg	XMG1.2	1:200	Biolegend
Anti-mouse-IL17A	eBio17B7	1:200	eBioscience
Anti-mouse-CD45RB	C363-16A	1:200	Biolegend
Anti-mouse-WASP	F-8	1:300	Santa Cruz Biotechnology
Anti-mouse-Dock8	G-2	1:500	Santa Cruz Biotechnology
Anti-mouse-STAT3	9139	1:1000	Cell Signaling
Anti-mouse-pSTAT3	4113	1:1000	Cell Signaling
Anti-mouse-STAT6	D3H4	1:1000	Cell Signaling
Anti-mouse-pSTAT6	Ab28829	1:1000	Abcam
Anti-mouse-pSTAT6	18/P-Stat6	1:20	BDBiosciences
Anti-human-CD4	A161A1	1:200	Biolegend
Anti-human-TNF	MAb11	1:200	Biolegend
Anti-human-IL10	3F9	1:50	Biolegend

Supplementary table 2: Primer sequence

Genes	Forward sequence	Reverse sequence
**Mouse**		
Hprt	GTTGGATACAGGCCAGACTTTGTTG	GAGGGTAGGCTGGCCTATAGGCT
Il10	CAAGGAGCATTTGAATTCCC	GGCCTTGTAGACACCTTGGTC
Ifng	ACTGGCAAAAGGATGGTGAC	TGAGCTCATTGAATGCTTGG
Il17a	GGCCCTCAGACTACCTCAAC	TCTCGACCCTGAAAGTGAAGG
Tnf	GACCCTCACACTCAGATCATCTTC	CTCCTCCACTTGGTGGTTTG
Il1b	GCAACTGTTCCTGAACTCAACT	ATCTTTTGGGGTCCGTCAACT
Nos2	GTTCTCAGCCCAACAATACAAGA	GTGGACGGGTCGATGTCAC
Il23p19	GACCCACAAGGACTCAAGGA	CATGGGGCTATCAGGGAGTA
Il12p35	TGGATCTGAGCTGGACCCTT	GCCAAAAAGAGGAGGTAGCG
Tgfbr1	TCCCAACTACAGGACCTTTTTCA	GCAGTGGTAAACCTGATCCAGA
Tgfbr2	CCGCTGCATATCGTCCTGTG	AGTGGATGGATGGTCCTATTACA
Tgfbr3	GGTGTGAACTGTCACCGATCA	GTTTAGGATGTGAACCTCCCTTG
Il10ra	AGGCAGAGGCAGCAGGCCCAGCAGA	TGGAGCCTGGCTAGCTGGTCACAGTA
Il10rb	GCTGCCTTCAGACTCTTC	AACCCCTCTGTGATCGGA
Arg1	CAGAAGAATGGAAGAGTCAG	CAGATATGCAGGGAGTCACC
Ym1	CTGAGAAGCTCATTGTGGGA	CTCAGTGGCTCCTTCATTCA
Fizz1	GTCCTGGAACCTTTCCTGAG	AGCTGGATTGGCAAGAAGTT
IL6	CTGCAAGAGACTTCCATCCAG	AGTGGTATAGACAGGTCTGTTGG
Socs3	GGGTGGCAAAGAAAAGGAG	GTTGAGCGTCAAGACCCAGT
IL4r	TCTGCATCCCGTTGTTTTGC	GCACCTGTGCATCCTGAATG
**Human**		
CXCL10	GTGGCATTCAAGGAGTACCTC	TGATGGCCTTCGATTCTGGATT
CCR7	TGAGGTCACGGACGATTACAT	GTAGGCCCACGAAACAAATGAT
CCL13	CTCAACGTCCCATCTACTTGC	TCTTCAGGGTGTGAGCTTTCC
SLC38A6	GCTTTTGACAGTCCCTCTAATCC	TTGATGTACTGGCACCAACTAC
MRC1	GGGTTGCTATCACTCTCTATGC	TTTCTTGTCTGTTGCCGTAGTT
CXCR4	ACGCCACCAACAGTCAGAG	AGTCGGGAATAGTCAGCAGGA
IL10	GACTTTAAGGGTTACCTGGGTTG	TCACATGCGCCTTGATGTCTG
IL6	ACTCACCTCTTCAGAACGAATTG	CCATCTTTGGAAGGTTCAGGTTG
TNF	GAGGCCAAGCCCTGGTATG	CGGGCCGATTGATCTCAGC
IL23p19	CAGCAACCCTGAGTCCCTAA	TCAACATATGCAGGTCCCACT
IL1β	AATCTGTACCTGTCCTGCGTGTT	TGGGTAATTTTTGGGATCTACACTCT
